# Medical Topography of Wharton, Texas

**Published:** 1861-04

**Authors:** Rowan Green

**Affiliations:** Wharton, Texas


### ARTICLE II.

*Abstract of an article on the Medical Topography of Wharton, Texas.* By Rowan Green, M. D., Wharton, Texas.

Having been in Wharton only ten months, I have not been able to become thoroughly acquainted with the climatic, geological, hydrographical and dietic influences brought to bear in the production of disease, and giving maladies the impress of peculiar characteristics found in this section of country. I hope, however, ere long, to avail myself of the offer of your pages not only to describe the face of the country, cause of disease, &c., but to present to the readers of the Journal the nature of the various diseases prevalent here and the plan of treatment found most successful.

Notwithstanding the large amount of immigration to this, as we say, "the garden spot of Texas," a considerable portion is yet wild and unsettled, owing to the very extensive prairies which spread themselves over a considerable portion of this country.

The land in this vicinity is exceedingly fertile, the rich alluvial soil extending to the depth of several feet. This super-stratum of the earth's surface is of a yellow brown color, intermixed with a considerable quantity of marine shells, and very adhesive.

Wharton county is in the 30th degree of latitude and about the 18th degree of longitude from Washington. It is about fifty miles from the gulf, about the same distance from Matagorda bay, and contains an area of 123,750 acres, and a population of 3,593 persons; of this number 2,798 are negro slaves. One year ago the negro population numbered 2,307, showing the annual increase of negroes at the rate of about 17 per cent. This of course does not depend on local increase entirely, but is to a considerable extent made up by immigration.

The face of the country, though undulating, is far from being what might be called "hilly." From a general and peculiar declivity in a southwesterly direction (probably owing to geological elevating agency in an opposite direction in past ages) the principal rivers, not only in this immediate section of the State, but throughout Western Texas, have, in process of time, been thrown westward of their original channels, as the face of the country plainly shows. This circumstance has much to do in laying the foundation for causes which probably now effect the health of this community, and we fear are to operate hereafter more powerfully in the production of malaria. The original channels thus deprived of their currents become chains of small lakes and pools of stagnant water, not particularly encouraging in a sanitary view of the country. On the Colorado, which runs through this county, and the Brazos, these lakes, on the supposed original beds of the rivers, are particularly conspicuous. Besides the Colorado, the country is watered by several constant, and some "winter and spring" streams. Some of these move sluggishly along through immense swamps of mud, while others are delightful little rivers, sending up no pestiferous fogs to becloud the horizon, or malarious poison to infect the peaceful tillers of their fertile banks.

The village of Wharton, with a population of not more than two hundred, is situated on the banks of the Colorado, and surrounded by the channels and low lands of this river on the west, and another small river or creek (old Caney), on the east. The latter is noted for the fertility of the soil, and dries up during summer.

The water from wells is more or less calcarious, and therefore unpleasant. Cisterns afford drinking water for the inhabitants. These are filled during the rainy seasons of the year and are so protected from the heat of the sun that this pleasant beverage is afforded throughout the warm seasons, of good quality. It is of course pure when properly kept.

The face of the country, away from the low lands is barren of trees. Extensive prairies stretch across the highlands between the streams, without a single tree to obstruct the view, for miles in the distance. To the botanist these are by no means uninteresting. Many medicinal plants here grow luxuriantly, and by the variety of their flowers give beauty to nature's garden. On the wood-land is found the ash, buckeye, cedar, cotton-wood, cypress, elm, hackberry, hickory, locust and pecan. Interspersed among these non-medicinal growths, my eye has fallen upon the following medicinal plants, without having given any special attention to the medicinal botany of this region: Aralia spinosa (prickly ash), rubus trivialis and rubus villosus, eupatorium, perfoliatum, burdock, cornus sericea (swamp dogwood), mustang grape, allium sativum, strammonium, cheuopodium anthelminticum, podophyllum, live oak, white oak, wild onion, amygdalis, persica (wild peachtree), prunus anericana, poke, ricinus communis, wild pepper, sumach, sarsaparilla, juglaus, salix, and some others.

The fertile prairie plains, which, in such large abundance, supply the *herbifera* with the richest of food, also, doubtless grow, luxuriantly, many plants known to be useful in curring the ailments of the *genus homo*. This is a fine field for the botanist, and should scientific examination be made of the medical botany of this country, there is no doubt it will be found rich in such productions.

